# Center of pressure characteristics from quiet standing measures to predict the risk of falling in older adults: a protocol for a systematic review and meta-analysis

**DOI:** 10.1186/s13643-019-1147-9

**Published:** 2019-09-07

**Authors:** Flavien Quijoux, Aliénor Vienne-Jumeau, François Bertin-Hugault, Marie Lefèvre, Philippe Zawieja, Pierre-Paul Vidal, Damien Ricard

**Affiliations:** 1 CNRS, UMR 8257 Cognition and Action Group, Paris, France; 2 Orpéa Group, Puteaux, France; 3 Hangzhou Dianzi University, Hangzhou, 310018 Zhejiang China; 4 Service de Neurologie de l’Hôpital d’Instruction des Armées de Percy, Service de Santé des Armées, Clamart, France; 5 Ecole du Val-de-Grâce, Service de Santé des Armées, Paris, France

**Keywords:** Older adults, Fallers, Quiet standing, COP, Prediction, Risk of falling

## Abstract

**Background:**

Falling is the most common accident of daily living and the second most prevalent cause of accidental death in the world. The complex nature of risk factors associated with falling makes those at risk amongst the elderly population difficult to identify. Commonly used clinical tests have limitations when it comes to reliably detecting the risk of falling, but existing laboratory tests, such as force platform measurements, represent one method of overcoming this lack of a test. Despite their widespread use, however, Center of Pressure (COP) signal analysis techniques vary and there is currently no consensus on which features should be used diagnostically. Our objective is to identify, through a systematic review and meta-analysis, the COP characteristics of older adults (≥ 60 years old) during quiet bipedal stance which will allow fallers to be distinguished from non-fallers.

**Methods:**

The systematic review will include both prospective and retrospective articles. Five databases will be searched: PubMed, Cochrane CENTRAL, EMBASE, and ScienceDirect. In addition, a search of gray literature will be performed using Google Scholar and ClinicalTrials.gov. Searches will be circumscribed to include only older adults (aged over 60 years) who underwent a bipedal quiet standing measure of their balance and for whom the number of falls was reported. Two authors will independently assess the risk of bias for each included article using a 26-item checklist. Funnel plots will be drawn to attest of possible publication biases for each COP parameters. The results will be synthesized descriptively and a meta-analysis will be undertaken. When trial methodological heterogeneity is too great for pooling of the data into a meta-analysis, evidence strength will be evaluated using best evidence analysis.

**Discussion:**

Despite the numerous advantages of posturography, the diversity of studies exploring balance in older fallers has led to uncertainty regarding the method’s ability to reliably identify fall-prone older adults. It is expected that the findings from this systematic review will help clinicians use bipedal quiet standing measures as a diagnostic test and allow researchers to explore COP characteristics to create better models for fall prevention care.

**Systematic review registration:**

PROSPERO CRD42018098671

**Electronic supplementary material:**

The online version of this article (10.1186/s13643-019-1147-9) contains supplementary material, which is available to authorized users.

## Background

The risk of falling amongst older people is a major healthcare issue representing one of the primary cause of injury and death in this demographic group [1]. In elderly people, even non-lethal falls can often lead to severe injuries such as hip fracture or traumatic brain injury [2–4]. Over 15% of people aged over 65 and 25% of those over 80 years will fall multiple times in the course of 1 year [5]. This risk and the consequences could be reduced by improving the screening tools used to detect fall-prone older people. At the time of writing, most commonly used clinical screening tools, such as the Timed Up and Go test (TUG), STRATIFY, Performance Oriented Mobility Assessment (POMA), and the Berg Balance scale (BBS), are retrospectively correlated to a patient’s fall history from previous months [6], but they have been repeatedly shown to lack both sensitivity and accuracy in order to be used prospectively to identify the fall-prone older adults [7–11]. Furthermore, these tests are also usually unable to follow changes in balance capacity with age in older people or the kind of change that occur in the early stages of neurological diseases [12]. The BBS, for example, requires an eight-point downgrade over 56 points in order to be meaningful [13, 14], and it is also prone to errors due to both floor and ceiling effects [15, 16]. Such drawbacks can also be seen in other tests whose subcomponents cannot be separated, which also makes them unsuitable for highly handicapped patients [17]. Other clinical tests, such as the STRATIFY tool and the TUG test, do not have well-defined thresholds for classifying patients as fallers or non-fallers. Due to these weaknesses, the predictive sensitivity and specificity of these tests are lowered [18]. As a consequence, they are more likely to be used as mere fall history questionnaires [11]. In addition to their subjective nature, results from clinical tests to evaluate balance need to be combined in order to identify the risk of falling [19]. Finally, even if the aforementioned tests are widely available in Gerontology Services, they cannot be used to discriminate fallers from non-fallers [20].

Quantitative posturographic tests, however, which assess balance by recording Center of Pressure (COP) oscillations [21] could provide a means to overcome these issues. The COP signal, usually assessed with force platforms, contains features that allow characterization of a patient’s postural strategies and modifications [22, 23]. Posturography also provides additional information on specific balance control mechanisms [24] and thus constitutes a clinically useful tool to identify those at risk of falling [25]. A better understanding of stabilization responses should therefore allow a more targeted management of the causes of imbalance in older people [26]. COP analysis has been used to determine motor strategies for fall prevention [27, 28], to reliably distinguish pathologies [29] and to link fear of falling with posturographic parameters [30]. Studies have indicated that some sway characteristics of a quiet stance, especially in the mediolateral direction, are significantly different between non-fallers and fallers and could therefore be good indicators of those at increased risk of future falls [31]. Amongst healthy, older adults who live in the community, balance and sway measurements have been shown to be strong predictors of fall risk [32, 33]. Despite this work, however, to date, there has been no study to summarize those COP features which best discriminate fallers from non-fallers amongst older people aged over 60. In 2006, Piirtola and Era [32] concluded that some COP parameters during bipedal quiet stance could help to predict risk of falls in the elderly. Unfortunately, the results of the nine articles included were contradictory and the measurement protocols used varied widely. Similarly, the narrative review by Pizzigalli et al. [31] reported some COP parameters as fall risk predictors. However, the contradictory results and the absence of quantitative analysis in these two articles limit the application of their conclusions in clinical practice. We hope that a more exhaustive literature search, and a quantitative study based on different recording protocols, will establish which parameters, and under what conditions, are associated with an increased risk of falling. We will seek to minimize protocol heterogeneity in order to draw conclusions that can be applied in practice. A bipedal quiet stance is a simple test to study balance motor strategy in older adults [28, 34] that, unlike unipedal or more complex tests, is more inclusive for an older population as it has a reduced incidence of participant exclusion due to falls during recording [35, 36]. Nevertheless, ways exist that make the test more challenging: one can add a double cognitive task [37], a soft support with a foam pad [38, 39] or asking the participant to close their eyes [40].

Therefore, the main aim of this systematic review is to extract the best biomarkers from COP bipedal quiet stance displacement data in order to (retrospective study) distinguish fallers from non-fallers and so (prospective study) predict fall risk. The second aim is to evaluate the accuracy of currently available predictive and classification models using these biomarkers.

### Objectives

This systematic review protocol was designed to address the following questions:

- Which features of the statokinesigram in older patients (≥ 60 years) during a bipedal quiet stance test differ between fallers and non-fallers?
- How well can the risk of falling in older adults be predicted from COP characteristics and analysis?
- Which parameters should be included in a predictive or a classification model of fall risk assessment for an older population?

## Methods

### Research protocol

This literature search and analysis was designed according to the PRISMA (Preferred Reporting Items for Systematic Reviews and Meta-Analyses) [41] and MOOSE (meta-analysis of observational studies in epidemiology) [42] guidelines. This protocol was registered in the PROSPERO database under the number CRD42018098671.

#### Search strategy

An electronic database search of titles and abstracts published will be performed between March 2017 and July 2019 to identify all articles published that include fall data for older people and their COP recordings. Five databases (PubMed, Cochrane CENTRAL, EMBASE, and ScienceDirect) will be used as sources for published articles. The search will be performed for articles published without date restriction until July 1, 2019, using associations of keywords (Table 1) from the PICO methodology. The following MeSH terms will be also used: “Accidental Falls/prevention & control,” “Accidental Falls/statistics & numerical data*,” “Aged,” “Postural Balance/physiology*,” “Posture/physiology*,” “Predictive Value of Tests,” and “Regression Analysis.” The main database search will be supplemented by a review of gray literature which will be conducted through web searches on Google Scholar and ClinicalTrials.gov. In addition, all reference lists and bibliographies of included studies will be themselves reviewed for relevant studies that were not picked up through any electronic search.

**Table 1:** Keywords from the P.I.C.O. framework

Components (apply AND for search)	Keyword used (apply OR for search)
Population	“OLDER ADULTS”
	“COMMUNITY-DWELLING PEOPLE”
	“ELDERLY”
	“SENIORS”
	“OUTPATIENT”
	“FALL PRONE ELDER”
	“NURSING HOME”
	“INSTITUTIONAL CARE”
Intervention	“BALANCE”
	“EQUILIBRIUM”
	“QUIET STANDING”
	“STANCE”
	“STANDING”
	“STABILITY”
	“POSTURE”
	“POSTURAL STABILITY”
Comparison	“POSTUROGRAPHY”
	“FALL*”
	“RISK OF FALLING”
	“CENTER OF PRESSURE”
	“CENTRE OF PRESSURE”
	“COP TRAJECTORY”
	“COP DISPLACEMENT”
	“SWAY”
	“STATOKINESIGRAM”
	“STABILOGRAM”
	“FORCE PLATFORM”
Outcomes	“PREDICT*”
	“DIAGNOS*”
	“CLASSIF*”
	“DISTING*”
	“DIFFERENC*”

#### Inclusion and exclusion criteria

Randomized control trials (RCTs), non-randomized control trials, and observational studies will all be eligible for inclusion. Due to the risk of bias arising from only including data from published RCTs [43, 44], data from gray literature will also be included provided that they have met the inclusion criteria (Table 2). Exclusion criteria will also be set (see Table 3).

**Table 2:** Inclusion criterion

Inclusion criterion domains	Explicit criterion
General criteria	- Published before July 1, 2018. - Related to the main topics: “the risk of falling in elderly people.” Articles not related to this topic will not be included based on the two-reviewer evaluation system.
Language criteria	- No language criteria are applied. However, for non-French, non-English, or non-Spanish articles, we will contact professional translators if no French, Spanish, or English version is found. Such translations will be indicated in the main article. - All full papers will be retrieved (or translated) and used.
Type-of-study criteria	- Retrospective and prospective clinical trials, randomized, or not. - Observational, time series, and cross-sectional studies.
Participants criteria	- Older patients (aged ≥ 60 years of age) considered to be otherwise healthy/without neurological disease as determined by a diagnostic assessment (or any specification from the authors) which could impact their posture including (but not limited to) Parkinson disease (PD), multiple sclerosis (MS), hemiplegia, paraplegic, stroke, or brain trauma. Orthopedic disorders affecting balance such as recent arthroplasty or amputation will also not be included in the review.
Intervention criteria	- Articles analyzing the balance through COP recordings during quiet standing with both feet on the ground and evaluating the risk of falling by the number of falls during a period of time (retrospectively or prospectively) - Any article measuring the risk of falling without an estimation of the number of falls per participant (i.e., indirect assessment through fear of falling tests or epidemiologic data only) or not related to the risk of falling (comparing elderly vs. young for example) will be discarded. - If training (e.g., exercise training or a physiotherapy program) is a part of the intervention, the article will be discarded unless a baseline of the quiet standing capacities is recorded. In this case, only the data from the baseline will be used.
Comparison criteria	- Fallers versus non-fallers (it can include “healthy elderly people” versus “fall prone elderly” or “low risk elderly” vs “high risk elderly” or “single fallers” versus “multiple fallers” or “infrequent fallers” versus “recurrent fallers”)
Outcomes criteria	- Primary outcomes will be the features in the COP analysis and their differences between the groups (odds ratio for dichotomous outcomes and mean differences for continuous outcomes). - Secondary outcomes will be the precision of the prediction (or the model) of the risk of falling, such as sensibility, specificity, area under the curve (AUC) of receiver operating characteristic (ROC) curves, number of true(/false) positive(/negative), positive predictive value (PPV), and negative predictive value (NPV), odd-ratio or other evaluation of the system.

**Table 3:** Exclusion criterion

Exclusion criterion domains	Explicit criterion
Human criteria	- All animal or pendulum-based studies will be discarded.
Intervention criteria	- All studies quantifying other activities than quiet standing (e.g., gait and equivalent, using a moving platform or moving environment for assessment, obstacle dodging, external destabilization, functional reach tests, one leg standing, or any forms of assessment of balance other than standing upright). - Romberg coefficient (difference between eyes opened and closed) will be accepted as well as standing on foam if there is a comparison with a firm surface. - Cognitive tasks which do not require to move (e.g., counting or memorizing) will be accepted. - A standardized posture is not an exclusion criterion but will be noted.
Outcome criteria	- A COP recording is mandatory to not be excluded. All studies than do not compute any parameter to quantify balance through COP data but focus on sway measurement only through sway meter, cumulative balance score (e.g., Sensory Organization Test) or motion capture will be discarded. Studies using Center of Mass (COM) without a COP recording will be discarded too.
Equipment criteria	- There are no equipment criteria as long as the research recorded COP displacement over time. Force platforms, pressure insoles, or any other COP recording systems are all accepted but will be noted.
Population criteria	- All studies including young (< 60 years old), healthy people without a comparison group of older people will be discarded. - The presence of a neurologic pathology that could influence posture will be an exclusion criterion. - All studies including recently post-operative participants will be discarded.
Comparison criteria	- All studies than do not compare elderly fallers and non-fallers but focus on methodological issues (e.g., COP features reliability, force platform methodology and validation, biomechanical model validation) will be discarded.

### Paper review process

Potentially eligible studies will be screened for inclusion eligibility independently by two review authors (FQ and AV) based on their title, abstract, and full text. Articles will first be imported into the Zotero® bibliographic database (Corporation for Digital Scholarship and the Roy Rosenzweig Center for History and New Media, USA) before screening so that all articles can be reviewed from the same source in order to select those that meet the criteria. If there is disagreement between the reviewers, the study will be discussed until a consensus is reached. Papers that are eligible will then be subjected to data extraction and a “risk of bias” evaluation, as described below.

#### Risk of bias evaluation

A quality/risk of bias assessment will be performed by using a 26-item checklist based on the work of Downs et al. [45] (Additional file 1). The checklist to be used will retain 18 items unchanged from the previous version of this checklist [46] while another three items will be removed and two extra items added. The final risk of bias assessment will also include a further six items from the original checklist that have been modified in order to evaluate the reliability of both the COP measures and the predictive models. In order to create and modify items, the Critical Appraisal Skills Programme (CASP) Evaluate a Clinical Prediction Rule Checklist (v13.03.17) will be used. Quality assessment for each article will be performed by two assessors (FQ and AV), and each assessor will be blind to the score given by the other until both have completed the evaluation. Any disagreement over the final score for each article will be discussed; if no agreement can be reached, the rounded mean of both scores will be used.

#### Data extraction and analysis

Following inclusion of the articles for analysis, the text from each reference will be imported into Microsoft Excel (version 2013, Microsoft Corp., Redmond, WA) for data extraction. One assessor (FQ) will extract and collate information following the recommendations of the Joanna Briggs Institute Reviewers’ Manual [47]. Another assessor (AV) will verify the extracted data from the included articles in order to confirm coherence of the data. Key characteristics to be extracted will include information about the study itself such as author(s), title, year of publication, inclusion and exclusion criteria, sample size, study methodology (retrospective or prospective fall evaluation), study duration, rate of falls, and mention of any adverse events that occurred during the study (Additional file 1). Population characteristics will also be recorded including demographic and biometric data such as participants’ gender, age, weight, height, BMI, and cognitive capacities (e.g., following a Mini Mental State Examination—MMSE). Data gathered about the falls will include the studies’ definition of a fall and how they were evaluated and the geographical location of the work (country, region, and establishment where the measures took place); quiet standing test parameters to be collected will include test conditions of the tests such as, for participants, whether they wore shoes or were barefoot, had their eyes open or closed, or if they were asked to use a comfortable or standardized foot position. For the test itself, data will be recorded on the type of standing surface (e.g., firm or foam) used, whether it was a cognitive double or single task, test duration, who performed the tests, the time interval between the different test parts, the data collection methods (type of tools, sampling frequency, and filter characteristics), and the COP features. For predictive (in prospective studies) or classification (in retrospectives studies) models, their characteristics and level of accuracy will also be extracted, when a statistical model has been used.

When these data are unavailable from the main text, Additional file 1 will be examined for more information. When data on the force platforms or other kind of equipment (such as the sampling frequency or the provider) are not available even in Additional file 1, the specifications will be sought from other articles by the same author(s). For experimental studies, the available COP data will be extracted from the baseline measurements that were taken before any intervention had been implemented as long as the history of fall is also available (retrospective classification). If the COP parameters before the intervention are not included, the article will not be analyzed. For observational studies with prospective evaluation of falls, data recorded before the follow-up assessment will used as in the analysis; if measurements were not performed before follow-up, the article will be excluded. Using software (like Plot Digitizer) to obtain data from figures was not considered as an option to extract data since this technique has been shown to be flawed concerning inter-rater reliability, with only a 50% agreement between both raters and an agreement of 70% with the original data even for trained raters [48]. In addition to the time consumption of extracting data by two authors independently, there is no guidance for this kind of extraction so far [49]. Therefore, we had rather not extracting the data on graphs to avoid introducing new biases.

Finally, authors will be contacted via e-mail up to three times to request missing data when they are not available in the main text or from other sources as described above.

### Strategy for data synthesis

Extracted data from included articles will be presented descriptively, especially study characteristics, population characteristics, COP features used, and the risk of bias. The risk of bias will be assessed using the value of the percentage scores from the 26-item checklist: score distribution will also be studied to look for a Gaussian distribution or, on the contrary, a trend in favor of the studies included in the meta-analysis. The quality scores will also be used as a parameter of the COP heterogeneity level in the meta-analysis.

For pooling predictor data from COP recordings, at least three studies must have used the same feature. If the included studies show consistency between their protocols, particularly with regard to the homogeneity of patient populations and the quiet standing test conditions, a meta-analysis of the aggregated data will be considered. For features that cannot be aggregated into a meta-analysis, a “best evidence synthesis” will be the preferred method of evaluating the strength of the studies’ evidence [50]. If data cannot be aggregated into a meta-analysis or if the results seem contradictory, the best evidence analysis will support articles with the highest score in the risk of bias assessment. Particular care will be taken to ensure that the methodological quality of the studies and consistency of their results are reported.

If a meta-analysis is indicated, the method will follow the Cochrane Collaboration handbook recommendations [51]. Means and standard deviations (SD) of measures will be used to compare the effect size of each parameter on the risk of falling and to allow the creation of forest plots. If SD data remain unavailable, even after contacting the authors, but standard errors or confidence intervals are available, we will calculate standard deviation values [29]. Effect size (ES) will be calculated using Eq. 1 [52, 53]:

1 $$ \mathrm{ES}=\left[1-\frac{3}{4\left({n}_1+{n}_2\right)-9}\right]\frac{\overline{y_1}-\overline{y_2}}{S} $$

ES is the unbiased effect size corrected for sample sizes *n*_1_ and *n*_2_ provided by Hedges; $\overline{y_1}$ and $\overline{y_2}$ are the means of each group and *S* is the pooled within-group standard deviation.

The estimated within-study variance of ES is computed from Eq. 2:

2 $$ {\hat{\sigma}}^2=\frac{n_1+{n}_2}{n_1{n}_2}+\frac{ES^2}{2\left({n}_1+{n}_2\right)} $$

Assuming a fixed-effects model, the weighting coefficient will be computed from Eq. 3:

3 $$ {\hat{w}}_{\mathrm{FE}}=1/{\hat{\sigma}}^2 $$

If a random-effects model is preferred, the weighting coefficient will be computed from Eq. 4:

4 $$ {\hat{w}}_{\mathrm{RE}}=1/\left({\hat{\sigma}}^2+{\hat{\tau}}^2\right) $$

5 $$ {\hat{\tau}}^2=\frac{Q-\left(k-1\right)}{c} $$

In Eq. 5, ${\hat{\tau}}^2$ is the estimated between-studies variance; *Q* is the heterogeneity statistic of the *k* independent studies and *c* the coefficient computed from Eq. 6:

6 $$ c=\sum {\hat{w}}_{\mathrm{FE}}-\frac{\sum \left({\hat{w}}_{\mathrm{FE}}\right)2}{\sum {\hat{w}}_{\mathrm{FE}}} $$

A fixed-effects model will be chosen if the heterogeneity is low to moderate (*I*^2^ < 50%) [54]; otherwise, a random-effect model will be used.

Finally, as shown in Eq. 7, the data will be pooled for meta-analysis in case of clinical, methodological, and statistical homogeneity to assess the mean effect size of a COP feature according to:

7 $$ \overline{\mathrm{ES}}=\frac{\sum \left(\hat{w}\times \mathrm{ES}\right)}{\sum \hat{w}} $$

### Confidence in cumulative evidence

Sensitivity analyses will explore the impact of recording settings on the COP results during the quiet standing measurement such as if patients had open or closed eyes, their foot position, standing surface firmness as well as whether the study was prospective or retrospective. The impact of COP measurement variability, due to factors like recording duration or sampling frequency [55], will also be discussed. Inter and intra-participant reliability for the different COP parameters will also be discussed in order to assess their usefulness in clinical practice [56–58]. If the data are detailed enough, the causes of falls will be investigated further to determine whether external factors independent of balance disorders were involved in the fall/non-fall status; such external factors could weaken the overall ability of COP measures to predict falls. If the heterogeneity for a given COP parameter within the meta-analysis is too great (as measured by *I*^2^ > 50%), the decrease of this heterogeneity will be tested by the deletion of studies that use a particular COP recording configurations (with a different material than the other studies included for this parameter for example); the heterogeneity decrease will then be discussed in relation to the study(s) deleted. If subgroups exist, e.g., recurrent fallers vs infrequent fallers, Microsoft Excel (ibid.) will be used for their analysis.

If enough RCTs and interventional studies can be included, the overall quality of the evidence for each outcome will be presented using the GRADE (Grading of Recommendations, Assessment, Development and Evaluation) criteria as per the Cochrane Collaboration [59]. Otherwise, the cumulative evidence will be assessed using our own rating system which is based on the GRADE system and was created to overcome the limitations of using GRADE on non-interventional observational studies. This system will give a score for each outcome based on (1) the mean risk of bias from every study included for that outcome, (2) the total number of studies used to pool the data, (3) a classification of heterogeneity from low to high, and (4) the overall sample size (Table 4). Each outcome could then be graded as either “High,” “Moderate,” “Low,” or “Very Low.”

**Table 4:** Cumulative evidence scale (low is rated 0, moderate 1, and high 2 for each item. The final rating is very low (< 2), low (2–4), moderate (5–6), high (> 6))

Quality	Risk of bias score (mean of the 32-score)	Number of studies (*n*)	Heterogeneity (*I*^2^)	Cumulative sample size	
High	> 22	> 10	< 30% (low heterogeneity)	> 400	
Moderate	16–22	3–10	30–75% (moderate)	200–400	
Low	< 16	0–3	> 75% (high heterogeneity)	< 200	
Score					Total

To visualize possible publication bias, funnel plots will be used to represent the estimated effect size of each article against the standard error mean plotted on the vertical axis. A symmetric inverted funnel shape suggests no publication bias. A funnel plot will be drawn for each COP parameter with respect to the type of study (retrospective or prospective).

## Discussion

This systematic review is expected to provide a valuable means of predicting and so preventing falls in older individuals by providing robust, evidence-based guidelines for the clinical and laboratory evaluation of risk of falling using a simple and reliable bipedal COP test.

The proposed study will retrieve and extract data from clinical trials and observational studies. It will report the spatio-temporal parameters of the center of pressure displacements during a bipedal quiet standing task in older people who are then classified as “fallers” and “non-fallers.” We have purposefully chosen bipedal tests because of the applicability of these tests to all older people. Unipedal tests, which are more difficult to perform, tend to exclude frailer individuals who find themselves unable to stand on one leg [60]. We do not think that conducting a sensitivity study based on this subgroup of people would be feasible due to a lack of individual data. We also chose to focus only on bipedal tests to reduce the diversity of recording methods used in the articles analyzed; including other methods for other tests would only further complicate the task of analyzing such already-heterogeneous data to obtain reliable results. Finally, we consider it possible that the motor strategies used to maintain balance during a one-legged stance are different from those used during bipedal stance [61] and, hence, a multivariate analysis of bipedal COP tests would be more suited as the topic of a separate, equally specific, systematic review.

Non-systematic reviews from other publications in this field have indicated that the reliability of the bipedal COP measurements appears to be high across the different study protocols [55, 62, 63] and it thus seems reasonable to assume that the repetition of measures will only increase this reliability. Biomechanical factors (such as height and weight) and acquisition settings are known to have a moderate to high influence on COP parameters [64, 65], and so particular attention will need to be paid to these factors in order to pool the data without bias.

One conceivable, and potentially major, limitation of this systematic review would be a lack of this participant and test protocol information in the included articles. In particular, fall circumstances can be key confounding variables: some COP measures might be associated with falls only under particular circumstances and not others. For parameters where the data are available, we will carry out a sub-analysis stratified by fall circumstances. We will also try to reduce these risks of bias by taking into account the quality of each study and by extracting information regarding the definition and evaluation of “a fall,” as well as data about adverse events gathered during the follow-up after from each acquisition protocol.

## Additional file

Additional file 1: 26-items quality checklist. Extracted data ordered by domain of interest. (DOCX 36 kb)

## Data Availability

Not applicable.

## Abbreviations

AUC: Area under the curve
BMI: Body mass index
COM: Center of mass
COP: Center of pressure
GRADE: Grading of Recommendations, Assessment, Development, and Evaluation
MMSE: Mini-mental state examination
MS: Multiple sclerosis
PD: Parkinson’s disease
RCT: Randomized control trial
ROC: Receiver operating characteristic
