# Supplementary material for: Center of pressure characteristics from quiet standing measures to predict the risk of falling in older adults: a protocol for a systematic review and meta-analysis
Source: Syst Rev. 2019 Sep 7;8:232. doi: 10.1186/s13643-019-1147-9 (PMC6731576; doi:10.1186/s13643-019-1147-9)
Supplement: Supplementary file 1 — 26-items quality checklist. Extracted data ordered by domain of interest. (DOCX 36 kb) [file 13643_2019_1147_MOESM1_ESM.docx]

# Additional file 1

# Supplemental material: 26-items quality checklist

**1. Reporting**

| 1. *Is the hypothesis/aim/objective of the study clearly described?* | | | |
| --- | --- | --- | --- |
| Yes | 1 | No | 0 |

| 1. *Are the main outcomes to be measured clearly described in the Introduction or Methods section?*   If the main outcomes are first mentioned in the Results, the question should be answered 'No'. | | | |
| --- | --- | --- | --- |
| Yes | 1 | No | 0 |

| 1. *Are the characteristics of the patients included in the study clearly described?*   In cohort studies and trials, inclusion and/or exclusion criteria should be given. In case-control studies, a case-definition and the source for controls should be given. We add the condition to report patients’ characteristics for the two subgroups of non-fallers and fallers to be scored as ‘Yes”. | | | |
| --- | --- | --- | --- |
| Yes | 1 | No | 0 |

| 1. *Is the COP recording clearly described?*   Equipment type, sampling frequency, duration, descriptions of the different conditions, standardized position (or not) and COP features are all included. | | | |
| --- | --- | --- | --- |
| Yes | 1 | No | 0 |

| 1. *Are the distributions of principal confounders in each group of subjects to be compared clearly described?*   A list of principal confounders is provided. Both acute confounders which can affect transitorily the statokinesigram during the evaluation (e.g. transitory low blood pressure, fatigue, etc.) and chronic states affecting the risk of falling (i.e., high BMI, cognitive impairment, history of falls, age over 80 years, proprioceptive loss, etc.) have been considered for this item. | | | | | |
| --- | --- | --- | --- | --- | --- |
| Yes | 2 | Partially | 1 | No | 0 |

| 1. *Are the main findings of the study clearly described?*   When they exist, quantitative findings should be reported in the ’results’ section and discussed in the ’discussion’ section for the main findings. | | | |
| --- | --- | --- | --- |
| Yes | 1 | No | 0 |

| 1. *Does the study provide estimates of the random variability in the data for the main outcomes?*   In non-normally distributed data the inter-quartile range of results should be reported. In normally distributed data the standard error, standard deviation or confidence intervals should be reported. If the distribution of the data is not described it must be assumed that the estimates used were appropriate and the questions should be answered ‘Yes’. | | | |
| --- | --- | --- | --- |
| Yes | 1 | No | 0 |

| 1. *Have all important adverse events that may be a consequence of the intervention been reported?* ***^¥^***   This should be answered ‘Yes’ if the study demonstrates that there was a comprehensive attempt to measure adverse events such as falls during evaluation. | | | |
| --- | --- | --- | --- |
| Yes | 1 | No | 0 |

| 1. *Have the characteristics of patients lost to follow-up been described?* ***^¥^***   This should be answered ‘Yes’ where there were no losses to follow-up or where losses to follow-up were so small that findings would be unaffected by their inclusion. This should be answered ‘No’ where a study does not report the number of patients lost to follow-up. | | | |
| --- | --- | --- | --- |
| Yes | 1 | No | 0 |

| 1. *Have actual probability values been reported (e.g. 0.035 rather than <0.05) for the main outcomes except where the probability value is less than 0.001?* | | | |
| --- | --- | --- | --- |
| Yes | 1 | No | 0 |

**2, External Validity**

*All the following criteria attempt to address the representativeness of the findings of the study and whether they may be generalised to the population from which the study subjects were derived.*

| 1. *Were the subjects asked to participate in the study representative of the entire population from which they were recruited?*   The study must identify the source population for patients and describe how the patients were selected. Patients would be representative if they comprised the entire source population, an unselected sample of consecutive patients, or a random sample. Random sampling is only feasible where a list of all members of the relevant population exists. Where a study does not report the proportion of the source populations from which the patients are derived, the question should be answered as ‘Unable to Determine’. | | | |
| --- | --- | --- | --- |
| Yes | 1 | No | 0 |
| Unable to Determine | 0 |  |  |

| 1. *Were those subjects who were prepared to participate representative of the entire population from which they were recruited?*   The proportion of those asked who agreed should be stated. Validation that the sample was representative would include demonstrating that the distribution of the main confounding factors was the same in the study sample and the source population. | | | |
| --- | --- | --- | --- |
| Yes | 1 | No | 0 |
| Unable to Determine | 0 |  |  |

| 1. *Was there a validation of the COP features used?*   If the COP-recording instrument is self-made and no analysis of reliability has been made in either the article or a precedent one, the score should be 0. If the COP features are self-computed and there is no validity analysis of these features in the present article or a precedent one, with references for instance, the score should be 0. | | | |
| --- | --- | --- | --- |
| Yes | 1 | No | 0 |
| Unable to Determine | 0 |  |  |

| 1. *Were the main outcomes be generalised to the entire population which the participants were recruited?*   If the authors used another group of similar patients to evaluate the out-of-sample difference of their results, the score should be 2. Train-test split or cross-validation procedure can be accepted as using another group. If the authors only compared the main outcomes with other authors’ studies, the score should be 1. If there is no attempt to generalize their findings, the score should be 0. | | | |
| --- | --- | --- | --- |
| With another group | 2 | Compared to other studies | 1 |
| No | 0 | Unable to Determine | 0 |

**3. Internal Validity – Bias**

| 1. *Has a definition of the fall been given with references?*   For studies where the number of falls would have been recorded with a proper definition based on references, this should be answered ‘Yes’. | | | |
| --- | --- | --- | --- |
| Yes | 1 | No | 0 |

| 1. *Was an attempt made to blind the results for the analysis?* ***^¥^***   *If the people who passed the tests are the same than those who made the analysis, this item should be answered ‘No’. If there is no precision about who made the analysis, this item should be answered ‘Unable to Determine’.* | | | |
| --- | --- | --- | --- |
| Yes | 1 | No | 0 |
| Unable to Determine | 0 |  |  |

| 1. *If any of the results of the study were based on ‘data dredging,’ was this made clear?*   Any analyses that had not been planned at the outset of the study should be clearly indicated. If no retrospective unplanned subgroup analyses were reported, then answer ‘Yes’. | | | |
| --- | --- | --- | --- |
| Yes | 1 | No | 0 |
| Unable to Determine | 0 |  |  |

| 1. *In trials and cohort studies, do the analyses adjust for different lengths of follow-up of patients, or in case-control studies, is the time period between the intervention and outcome the same for cases and controls?* ***^¥^***   Where follow-up was the same for all study patients the answer should be ‘Yes’. If different lengths of follow-up were adjusted for by, for example, survival analysis, the answer should be ‘Yes’. Studies where differences in follow-up are ignored should be answered ‘No’. | | | |
| --- | --- | --- | --- |
| Yes | 1 | No | 0 |
| Unable to Determine | 0 |  |  |

| 1. *Were the statistical tests used to assess the main outcomes appropriate?*   The statistical techniques used must be appropriate to the data. For example, non-parametric methods should be used for small sample sizes. Where little statistical analysis has been undertaken but where there is no evidence of bias, the question should be answered ‘Yes’. If the distribution of the data (normal or not) is not described it must be assumed that the estimates used were appropriate and the question should be answered ‘Yes’. | | | |
| --- | --- | --- | --- |
| Yes | 1 | No | 0 |
| Unable to Determine | 0 |  |  |

| 1. *Was compliance with the falls measurement reliable?****^¥^***   Where there was non-compliance with the self-measured number of fall or no attempt to contact the participants to recall their falls, the question should be answered ‘No’. For studies where the number of falls was measurement by professional care givers, the question should be answered ‘Yes’. | | | |
| --- | --- | --- | --- |
| Yes | 1 | No | 0 |
| Unable to Determine | 0 |  |  |

| 1. *Were the main outcomes of the study been reported?*   The number of falls and the COP features should be reported for each group or subgroup of participants. The outcomes for the classification or the validity of the model should be reported if there is a predictive model. ***N.B. This question does not cover statistical tests.*** | | | |
| --- | --- | --- | --- |
| Yes | 1 | No | 0 |
| Unable to Determine | 0 |  |  |

**4. Internal Validity – Confounding (Selection Bias)**

| 1. *Were the patients in different groups (trials and cohort studies) or were the cases and controls (case-control studies) recruited from the same population?*   For example, patients for all comparison groups should be selected from the same hospital, institution or community of people. Patients and controls should not have significantly different age. The question should be answered ‘Unable to Determine’ for cohort and case-control studies where there is no information concerning the source of patients included in the study. | | | |
| --- | --- | --- | --- |
| Yes | 1 | No | 0 |
| Unable to Determine | 0 |  |  |

| 1. *Were study subjects in different intervention groups (trials and cohort studies) or were the cases and controls (case-control studies) recruited over the same period of time?*   For a study which does not specify the time period over which patients were recruited, the question should be answered ‘Unable to Determine’. | | | |
| --- | --- | --- | --- |
| Yes | 1 | No | 0 |
| Unable to Determine | 0 |  |  |

| 1. *Was there adequate adjustment for confounding in the analyses from which the main findings were drawn*?   This question should be answered ‘No’ for trials if the main conclusions of the study were; i) only based on the analysis of the participants’ data whom complete the follow-up period instead of whole population and adjusting the results for the follow-up duration; ii) the distribution of known confounders in the different groups was not described; or iii) the distribution of known confounders differed between the groups but was not taken into account in the analyses. Both acute and chronic confounders can be used for adjustment.  In non-randomised studies if the effect of the main confounders was not investigated or confounding was demonstrated but no adjustment was made in the final analyses the question should be answered ‘No’. | | | |
| --- | --- | --- | --- |
| Yes | 1 | No | 0 |
| Unable to Determine | 0 |  |  |

| 1. *Were losses of patients to follow-up taken into account?* ***^¥^***   If the number of patients lost to follow-up is not reported, the question should be answered as ‘Unable to Determine’. If the proportion lost to follow-up was too small to affect the main findings, the question should be answered ‘Yes’. | | | |
| --- | --- | --- | --- |
| Yes | 1 | No | 0 |
| Unable to Determine | 0 |  |  |

**5. Power**

| 1. *Did the study have sufficient power to detect a clinically important effect where the probability value for a difference being due to chance is less than 5%?*   Sample sizes have been calculated to detect a different of x% and y%. | | | |
| --- | --- | --- | --- |
|  | Size of smallest intervention group | Power Estimate | Score |
| A | < n_1_ | 70% | 0 |
| B | n_1_-n_2_ | 80% | 1 |
| C | n_3_-n_4_ | 85% | 2 |
| D | n_5_-n_6_ | 90% | 3 |
| E | n_7_-n_8_ | 95% | 4 |
| F | n+ | 99% | 5 |

# Supplemental material: Extracted data ordered by domain of interest

| Extracted data domain |  |
| --- | --- |
| General criteria | - Authors - Title - Year of publication - Geographical location of the study |
| Type-of-study criteria | - Inclusion/Exclusion criteria - Retrospective *or* Prospective evaluation of fall - Consecutive *or* random inclusion - Period that study was carried out - Definition of the fall - Evaluation of the fall |
| Participant criteria | - Mean age - Weight - Height - Body Mass Index - Cognitive test results (especially Mini Mental State Examination –MMSE-) - Sex ratio - Sample size - Living arrangements (i.e. living independently, living in the community, regular medical establishment appointments, outpatient, nursing home, inpatient, group of patients with divers pathologies, mix of profiles, not specified) |
| Intervention criteria | - Patient condition (i.e. barefoot/with shoes, feet position, eyes open/closed) - Type of surface (firm or foam) - Simple *or* dual task - Test duration - Data collection method: measurement tool, sample frequency, resampling, type and cut-off frequency for the filter - Time interval between the different tests |
| Comparison criteria | - Faller/Non-faller categorization: based on number of falls - Test personnel: number, role, training and expertise - Fall rate during follow-up - Adverse events |
| Outcome criteria | - Measured COP features - Type of modeling for prediction or classification - Validation of model |
